# Impaired Sequential Working Memory in Patients With Young Onset Parkinson's Disease

**DOI:** 10.1002/brb3.70182

**Published:** 2024-12-06

**Authors:** Guanyu Zhang, Shuo Zhang, Zhenzhen Zhao, Jinghong Ma, Piu Chan, Zheng Ye

**Affiliations:** ^1^ China Institute of Sport Science Beijing China; ^2^ Department of Neurology Taian Hospital of Traditional Chinese Medicine Taian China; ^3^ Department of Geriatrics Center The Fourth People's Hospital of Shenyang Shenyang China; ^4^ Department of Neurology Xuanwu Hospital of Capital Medical University Beijing China; ^5^ Department of Neurobiology, Neurology and Geriatrics, Beijing Institute of Geriatrics Xuanwu Hospital of Capital Medical University Beijing China; ^6^ Institute of Neuroscience, Center for Excellence in Brain Science and Intelligence Technology Chinese Academy of Sciences Shanghai China

**Keywords:** akinetic rigid type, dopamine D2/3 receptor agonists, neuropsychological tests, sequential working memory, young onset Parkinson's disease

## Abstract

**Background:**

Sequential working memory refers to the cognitive ability to maintain and/or manipulate a set of ordered representations within a short period. It remains unclear whether sequential working memory is impaired in patients with young onset Parkinson's disease (YOPD).

**Objectives:**

The aim of this study is to evaluate the sequential working memory in patients with YOPD.

**Methods:**

Sixty‐three YOPD patients (29 women) and one hundred age‐ and education‐matched healthy controls participated in three well‐established sequential working memory tests. The YOPD patients were categorized into akinetic rigid type (PD‐ART) and non‐akinetic rigid type (PD‐NART). Participants were asked to maintain digit sequences in mind in the digit span forward (DST‐F) and to maintain and manipulate digit sequences in mind in the digit span backward (DST‐B) and adaptive digit ordering tests (DOT‐A).

**Results:**

The PD‐ART group scored lower and had higher ordering costs (difference between the DST‐F and DOT‐A scores) than the healthy control group in the DOT‐A. Moreover, in the PD‐ART group, the daily levodopa equivalent dose for dopamine D2/3 receptor agonists positively correlated with the DOT‐A score and negatively correlated with the DOT‐A ordering cost, suggesting that patients who took a greater dose of dopamine D2/3 receptor agonists tended to have higher DOT‐A scores and lower DOT‐A ordering costs.

**Conclusions:**

These results indicated that the impaired sequential working memory may be one of markers of identifying early cognitive impairment in patients with YOPD, especially in PD‐ART patients. The dopamine D2/3 receptor agonists can recover this impairment to some extent.

## Graphical abstract

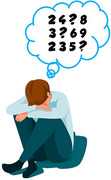

## 1 Introduction

Parkinson's disease (PD) is the most common movement disorder characterized by resting tremor, bradykinesia, postural instability, and muscle rigidity (Han et al. 2012). Because of the heterogeneous nature of PD, it can be classified into distinct subtypes: akinetic rigid type (PD‐ART), tremor dominant type (PD‐TDT), and mixed type (PD‐MT) (Kang et al. 2005). Importantly, the different subtypes exhibit variability of non‐motor symptoms. For example, patients with PD‐ART showed greater cognitive decline and affective dysfunction than PD‐TDT patients (Xu et al. 2018).

Most PD patients develop the disease around age 60, that is, typical onset PD (TOPD). The incidence of early onset PD is 1.5/100,000 per year in those aged under 50 years (Wickremaratchi, Ben‐Shlomo, and Morris 2009). Individuals who were diagnosed at age 21–50 are defined as young onset PD (YOPD) (Xu et al. 2018). Compared to TOPD, YOPD patients have a slower progression, higher incidence of levodopa‐induced dyskinesia, less disability, and lower prevalence of dementia (Schrag and Schott 2006). Thus, it's meaningful to identify effective markers of YOPD for early intervention.

Sequential working memory is the cognitive ability to maintain and/or manipulate a series of ordered information within a short period of time (Wickremaratchi, Ben‐Shlomo, and Morris 2009). PD patients have poor performance in organizing the thoughts and actions in a specific order with certain rules. For example, PD patients at early stages have difficulty in comprehending the temporal relation of events expressed without their actual order of occurrence and arranging digit sequences (Ye et al. 2012; Holden, Schweer, and Tröster 2021). There were relatively fewer studies on pure YOPD. Most of them focused on TOPD or included both YOPD and TOPD patients (Zhang et al. 2021; Schrag and Schott 2006), so it's unclear that whether the impairments in sequential working memory occur in pure YOPD.

Dual‐state theory pointed out that dopamine regulates the working memory by D1‐ and D2‐class receptors (Durstewitz and Seamans 2008). Concretely, D1‐dominated state facilitates persistent maintenance of items and D2‐dominated state is benefit for flexible manipulation of items. It's been confirmed that dopamine D2/3 receptor agonists play a role on ordering time in sequential working memory in PD patients (Zhang et al. 2021). Therefore, it's interesting to verify whether this effect also exists in pure YOPD patients.

In clinical practice, three standardized neuropsychological tests with oral responses are readily used for assessing sequential working memory. The digit span test (DST) is extracted from the Wechsler Adult Intelligence Scale, which is composed of the digit span forward test (DST‐F) and digit span backward test (DST‐B) (Wechsler, 1945; Jasinski et al. 2011). As a simple, quick, and inexpensive test, the DST can help to distinguish between PD and progressive supranuclear palsy patients at early stages (Ye et al. 2012). Moreover, DST has been confirmed to predict mild cognitive impairment with high diagnostic validity in PD (Biundo et al. 2013). The adaptive digit ordering test (DOT‐A) (Werheid et al., 2002), a updated version of the digit ordering test developed in analogy to the DST (Cooper et al. 1991), is defined as a useful diagnostic tool because of its similarity and comparability to the DST and high efficiency and sensitivity for PD patients.

In this study, generally, we aimed to investigate the sequential working memory of YOPD patients. All participants completed three well‐established sequential working memory tests, including DST‐F, DST‐B, and DOT‐A. We used the DST‐F to assess the maintenance capacity of sequential digits, and the DST‐B and DOT‐A to assess the maintenance and manipulation capacity of sequential digits. Specifically, we investigated group differences in test scores and ordering costs (difference between DST‐F and DST‐B/DOT‐A scores) and explored the role of dopamine D2/3 receptor agonists on test scores and ordering costs by partial correlation tests.

## 2 Methods

This study was approved by the research ethics committee of the Capital Medical University Xuanwu Hospital following the Declaration of Helsinki. The signed written informed consent was obtained from each subject before taking part in this study.

### 2.1 Patients and Clinical Assessments

We recruited 63 YOPD patients diagnosed following Movement Disorder Society Clinical Diagnostic Criteria (Postuma et al., 2015) at the Capital Medical University Xuanwu Hospital between 2023 and 2024. Inclusion criteria were (1) Hoehn and Yahr Stages 1 to 2.5; (2) native Chinese speakers; (3) age at onset 21 to 50 years; and (4) education ≥5 years. Exclusion criteria were (1) drug or alcohol abuse; (2) a history of brain lesions, other psychiatric or neurological diseases; (3) possible dementia or mild cognitive impairment (Montreal Cognitive Assessment, MoCA<26/30) or intake of anti‐dementia drugs; (4) possible depression (Beck Depression Inventory‐II, BDI‐II>7) or receiving depression treatment. The depression is temporally associated with cognitive impairment (Wang and Blazer 2015).

The Movement Disorder Society‐sponsored revision of the Unified Parkinson's Disease Rating Scale (MDS‐UPDRS) Part I and Part III subscales were used to evaluate the severity of non‐motor and motor symptoms, respectively. The MDS‐UPDRS has four subscales, namely, Part I: Non‐motor Experiences of Daily Living; Part II: Motor Experiences of Daily Living; Part III: Motor Examination; Part IV: Motor Complications (Goetz et al. 2008). All questions from these four subscales were designed to a questionnaire format for patient/caregiver and investigator. Each question has five options that are associated with commonly accepted clinical terms: 0 = normal, 1 = slight, 2 = mild, 3 = moderate, and 4 = severe. The REM Sleep behavior disorder screening questionnaire and Epworth Sleep Scale were used to assess motor behaviors during REM sleep and daytime sleepiness, respectively. PD patients were identified into PD‐ART (*N* = 37), PD‐TDT (*N* = 7), and PD‐MT (*N* = 19) on the basis of their MDS‐UPDRS Part III scores (Lewis et al. 2005). Given the sample size, we defined the PD‐TDT and PD‐MT groups as the non‐PD‐ART (PD‐NART) group. All patients were evaluated on their regular anti‐parkinsonian drugs, including levodopa (*N* = 17 for PD‐ART; *N* = 10 for PD‐NART), pramipexole (*N* = 15 for PD‐ART; *N* = 8 for PD‐NART), amantadine (*N* = 11 for PD‐ART; *N* = 1 for PD‐NART), piribedil (*N* = 5 for PD‐ART; *N* = 6 for PD‐NART), selegiline (*N* = 5 for PD‐ART; *N* = 0 for PD‐NART), and rasagiline (*N* = 0 for PD‐ART; *N* = 1 for PD‐NART). The total daily levodopa equivalent dose was calculated according to the method published by Tomlinson et al. (2010). The daily levodopa equivalent dose for dopamine D2/3 receptor agonists was defined as the sum of daily levodopa equivalent dose of drugs belonging to dopamine D2/3 receptor agonists (e.g., pramipexole, piribedil). Table 1 demonstrates demographic, clinical, and neuropsychological assessments.

**TABLE 1:** Demographic, clinical, and neuropsychological assessments of patients and healthy controls (means, standard deviations, and group differences).

Features/measures	Akinetic rigid type (*N* = 37)	Non‐akinetic rigid type (*N* = 26)	Healthy controls (*N* = 100)	Group differences (*p* Values)
Male:female	19:18	15:11	48:52	0.673
Age at evaluation (years)	44.5 (6.3)	44.8 (6.7)	44.8 (15.3)	0.992
Education (years)	13.1 (3.2)	14.1 (3.5)	13.5 (3.1)	0.505
Disease duration (years)	2.9 (2.1)	2.7 (2.3)	—	0.750
*Motor symptoms*				
MDS‐UPDRS III: Motor Examination	23.5 (13.6)	21.2 (13.5)	—	0.508
Akinetic‐rigid score	11.2 (6.1)	6.6 (5.2)	—	0.003
Tremor score	1.2 (1.4)	4.2 (2.8)	—	<0.001
Hoehn and Yahr Scale	1.6 (0.5)	1.4 (0.5)	—	0.274
Duration of motor symptoms (years)	3.1 (2.1)	3.0 (2.8)	—	0.868
*Daily levodopa equivalent dose*				
Total (mg/day)	253.8 (248.2)	188.0 (235.6)	—	0.301
Dopamine D2/3 receptor agonists (mg/day)	43.0 (52.8)	56.0 (63.0)	—	0.381
*Non‐motor functions*				
MDS‐UPDRS I: Non‐motor experiences of daily living	3.9 (3.6)	3.7 (2.7)	—	0.801
Beck Depression Inventory‐II	2.8 (2.1)	2.9 (2.4)	2.3 (2.1)	0.313
REM Sleep behavior disorder screening questionnaire	7.4 (8.2)	7.2 (12.2)	5.6 (7.1)	0.503
Epworth Sleep Scale	3.0 (2.9)	2.0 (2.7)	3.8 (2.4)	0.020
Montreal Cognitive Assessment	28.0 (1.3)	28.0 (1.4)	27.6 (1.4)	0.266
*Sequential working memory tests*				
Digit span forward test score	8.5 (1.2)	8.0 (1.2)	8.6 (1.2)	0.045
Digit span backward test score	5.6 (1.6)	5.1 (1.6)	5.8 (1.6)	0.099
Adaptive digit ordering test score	6.7 (2.1)	7.4 (2.4)	7.9 (2.0)	0.008
Digit span backward test ordering cost	2.9 (1.4)	2.8 (1.5)	2.8 (1.4)	0.911
Adaptive digit ordering test ordering cost	1.8 (1.9)	0.9 (2.6)	0.7 (1.8)	0.010

*Note*: MDS‐UPDRS, the Movement Disorder Society‐sponsored revision of the Unified Parkinson's Disease Rating Scale; Group differences, *p* values of Kruskal–Wallis one‐way ANOVAs, one‐way ANOVAs, or one‐way ANCOVAs as appropriate.

### 2.2 Healthy Control Group

We recruited 100 age‐ and education‐matched healthy controls (HC). Exclusion criteria were (1) drug or alcohol abuse; (2) a history of any psychiatric or neurological diseases; (3) possible current mild cognitive impairment or dementia (MoCA<26/30); (4) possible depression at last assessment. They completed the same assessments for cognition, sleep, and emotion as patients.

### 2.3 Sequential Working Memory Tests

All subjects were assessed by three sequential working memory tests, including the DST‐F, DST‐B, and DOT‐A. They heard a series of random digits at speed of one digit per second in each trial by a well‐trained experimenter and immediately recalled the digits in original order in DST‐F, in reversed order in DST‐B, and in ascending order in DOT‐A. Each test started at a 3‐digit trial and was terminated when participants failed to recall the digits in both trials of a certain length. For example, the experimenter started to read 3‐7‐2 at speed of one digit per second. If the participant correctly responded, the experimenter would read 7‐4‐5‐3. If the participant incorrectly responded, the experimenter would read 8‐6‐7‐4. If the participant still incorrectly responded, the test would be terminated. In the DST‐F and DST‐B, if the participant correctly recalled the first trial of a certain length, then the experimenter will read the first trial of the next length. In the DOT‐A, the experimenter read the second trial of a certain length even if the participant correctly recalled the digits in the first trial. Given the difference in practice, we chose different scoring rules to increase comparability. For the DST‐F and DST‐B, we scored the length of final successfully recalled trial (span). For the DOT‐A, we scored the count of successfully recalled trials.

### 2.4 Statistical Analysis

Data were analyzed by IBM SPSS Statistics 20. First, we detected group differences in test scores and ordering costs by one‐way ANCOVAs (two‐tailed, *p* < 0.010 Bonferroni correction for five tests). The DST‐B ordering cost was defined as DST‐F score minus DST‐B score and the DOT‐A ordering cost was defined as DST‐F score minus DOT‐A score (Ma et al. 2018). The ANCOVA had a factor Group (HC, PD‐ART, PD‐NART), and covariates Sex, Age, and Education. Significant differences were accompanied by two‐sample *t*‐tests.

Given the significant differences in DOT‐A score and ordering cost, second, we examined the role of dopamine D2/3 receptor agonists by correlating the daily levodopa equivalent dose for dopamine D2/3 receptor agonists with DOT‐A score and ordering cost in PD‐ART and PD‐NART, respectively (two‐tailed, *p* < 0.013 Bonferroni correction for four tests). In the partial correlation tests, the daily levodopa equivalent dose for other drugs (difference between the total daily levodopa equivalent dose and daily levodopa equivalent dose for dopamine D2/3 receptor agonists), Sex, Age, and Education were defined as control variables.

Our sample size could achieve 0.85 statistical power for all analyses using Power Analysis and Sample Size software (Bujang and Adnan 2016).

## 3 Results

### 3.1 Group Differences in Test Scores and Ordering Costs

**FIGURE 1:**
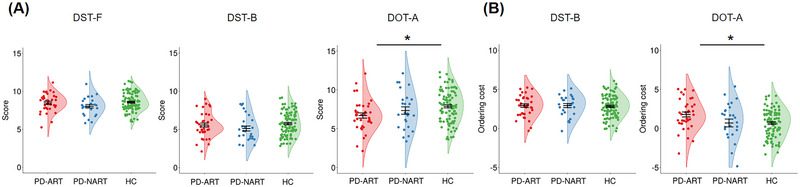
(A) Individual data, group means, and standard errors of digit span forward test (DST‐F), digit span backward test (DST‐B), and adaptive digit ordering test (DOT‐A) scores in Parkinson's disease patients with akinetic rigid type (PD‐ART), with non‐akinetic rigid type (PD‐NART), and healthy controls (HC). Asterisks, *p* < 0.05. (B) Individual data, group means, and standard errors of DST‐B and DOT‐A ordering costs in each group. Asterisks, *p* < 0.05.

Table 1 and Figure 1A demonstrate test scores in each group. Significant group difference was found in the DOT‐A score (*F*(2, 157) = 5.00, *p =* 0.008, η*_p_* ^2^ = 0.06), but not in the DST‐F (*F*(2, 157) = 3.17, *p =* 0.045, η*_p_* ^2^ = 0.04) or DST‐B score (*F*(2, 157) = 2.35, *p =* 0.099, η*_p_* ^2^ = 0.03). The PD‐ART group, but not the PD‐NART, scored lower than the HC group in the DOT‐A (*t*(135) = ‐3.04, *p =* 0.003).

Table 1 and Figure 1B demonstrate ordering costs in each group. Significant group difference was found in the DOT‐A ordering cost (*F*(2, 157) = 4.70, *p =* 0.010, η*_p_* ^2^ = 0.06) but not in the DST‐B ordering cost (*F*<1). The PD‐ART group had larger DOT‐A ordering costs than the HC group (*t*(135) = 3.14, *p =* 0.002).

It means that PD‐ART patients’ ability to manipulate digits was impaired especially in the ascending rule. Their maintenance ability was preserved.

### 3.2 Role of Dopamine D2/3 Receptor Agonists on DOT‐A Score and Ordering Cost

**FIGURE 2:**
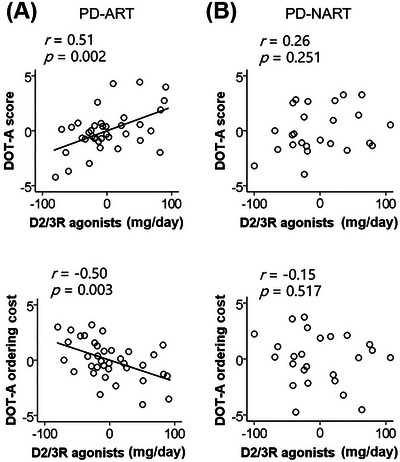
(A) In PD‐ART, the daily levodopa equivalent dose for dopamine D2/3 receptor agonists positively correlated with DOT‐A score and negatively correlated with DOT‐A ordering cost when the daily levodopa equivalent dose for other drugs, Sex, Age, and Education were controlled. (B) In PD‐NART, there was no correlation between the daily levodopa equivalent dose for dopamine D2/3 receptor agonists and DOT‐A score or ordering cost. The unstandardized residuals of data were employed and values were demeaned.

Figure 2A shows the role of dopamine D2/3 receptor agonists on DOT‐A score and ordering cost in PD‐ART. We found that the daily levodopa equivalent dose for dopamine D2/3 receptor agonists positively correlated with DOT‐A score (*r* = 0.51, *p* = 0.002) and negatively correlated with DOT‐A ordering cost (*r* = ‐0.50, *p* = 0.003) when the daily levodopa equivalent dose for other drugs, Sex, Age, and Education were controlled. The patients who took a greater dose of dopamine D2/3 receptor agonists tended to have higher DOT‐A scores and lower DOT‐A ordering costs.

In PD‐NART (Figure 2B), we did not find that the correlation between daily levodopa equivalent dose for dopamine D2/3 receptor agonists and DOT‐A score or ordering cost (*p*s≥0.251) when the daily levodopa equivalent dose for other drugs, Sex, Age, and Education were controlled.

Dopamine D2/3 receptor agonists may be beneficial to manipulate sequences in PD‐ART patients.

## 4 Discussion

In this study, we assessed sequential working memory in YOPD patients. We found that PD‐ART patients scored lower in DOT‐A and had higher DOT‐A ordering costs than the healthy adults. In other words, the maintenance capacity was preserved but the manipulation capacity was impaired in PD‐ART, especially in the ascending order rule. Importantly, in PD‐ART, the daily levodopa equivalent dose for dopamine D2/3 receptor agonists positively correlated with DOT‐A score and negatively correlated with DOT‐A ordering cost, suggesting that dopamine D2/3 receptor agonists may be beneficial for the manipulation of sequences in PD‐ART patients.

In previous studies, more attention was paid to psychosocial implications rather than cognition for YOPD patients (Willis et al. 2013). This study proposed that YOPD patients exhibited a selective impairment in manipulation of sequences, especially in PD‐ART patients but not PD‐NART patients, which may be due to pathological differences between different phenotypes. Biomarker studies pointed out that PD‐ART patients represent more diffuse and advanced neurodegeneration than PD‐TDT patients, involving dopaminergic and noradrenergic as well as synuclein and amyloid pathologies (Marras, and Chaudhuri 2016).

Tokuhara, Fujita, and Kashimori. (2021) tried to elucidate the mechanisms of the maintenance and manipulation of working memory. They used a delayed match‐to‐category task and reported that the sequence information is maintained in a persistent activity of a subarea and is manipulated by relearning the association between another subarea encoding sequential information and a decision area. Moreover, the prefrontal cortex provides a basis for this mechanism. D'Esposito et al. (1999) designed a delayed‐response task, in which participants were asked to hold a series of letters after the delay stage (maintenance) or reorder the letters in the alphabetical order after the delay stage (manipulation). They found that dorsolateral prefrontal cortex was more activated in manipulation trials than in maintenance trials.

A dynamic gating mechanism proposed by Hazy, Frank, and O'Reilly. (2006) argues how to switch between updating and maintenance. When the gate is open for new information, current working memory content can acquire updating; when it is closed to inhibit irrelevant information, working memory content can be robustly maintained. The basal ganglia contribute significantly to this dynamic gating mechanism. Furthermore, a frontobasal ganglia mechanism for sequential working memory proposed that sequence manipulation is realized by the interaction between basal ganglia and the prefrontal cortex in PD patients (Ye et al., 2021). The more serious manipulation impairment in PD‐ART patients may be due to decreased activation of the prefrontal cortex and basal ganglia compared to PD‐TDT patients and healthy adults (Prodoehl et al., 2013). Future studies should examine the distinction between PD‐ART and PD‐NART patients in functional anatomy of frontal‐basal ganglionic circuits and sequence manipulation‐induced activity by neuroimaging.

Neurochemical underpinnings of sequential working memory remain unclear. Our results were consistent with previous studies. It has been proven that dopamine D2 receptor agonist bromocriptine was benefit to improve retrieval efficiency in verbal working memory (Gibbs and D'Esposito 2005). The PD patients who took a greater dose of dopamine D2/3 receptor agonists tended to have lower error rates in DOT‐A (Ma et al., 2019). However, a review pointed out that larger daily levodopa equivalent dose was associated with working memory deficit in PD patients (Ramos and Machado, 2021). This inconsistency could be explained by the dopamine overdose hypothesis (Vaillancourt et al., 2013). The inverted‐U shaped the relationship between dopamine levels and cognitive performances in PD patients. Individual gene polymorphisms and dopaminergic pharmacotherapy can affect this inverted‐U dose‐performance relationship. Due to these contributory factors, the patients can respond negatively or positively to dopaminergic medicine when performing cognitive tasks (e.g., reversal learning and motor sequence learning tasks). In addition, this study recruited mild PD patients with relatively small daily levodopa equivalent dose, which did not reach the inflection point of the inverted U‐shaped curve, so the effect of dopamine D2/3 receptor agonists on sequential working memory is positive.

This study has limitations. First, consensus is lacking concerning the age definition for YOPD (Pagano et al., 2016). We chose a more common classification method and made the sample size as large as possible. Second, no causal conclusion can be drawn about the role of dopamine D2/3 receptor agonists on sequential working memory in YOPD. Future pharmacological studies can realize this issue.

## 5 Conclusion

In this study, we assessed the sequential working memory in YOPD patients by three well‐established neuropsychological tests. YOPD patients’ sequence maintenance capacity was preserved, but their manipulation capacity was impaired, especially in PD‐ART patients. Importantly, dopamine D2/3 receptor agonists may have a positive effect on the manipulation of sequences in PD‐ART.

## Author Contributions

**Guanyu Zhang**: formal analysis, funding acquisition, writing–original draft, visualization. **Shuo Zhang**: investigation, writing–review and editing. **Zhenzhen Zhao**: investigation, writing–review and editing. **Jinghong Ma**: investigation, writing–review and editing. **Piu Chan**: funding acquisition, writing–review and editing. **Zheng Ye**: conceptualization, funding acquisition, writing–review and editing.

## Conflicts of Interest

The authors declare no conflicts of interest.

### Peer Review

The peer review history for this article is available at https://publons.com/publon/10.1002/brb3.70182.

## Data Availability

The data employed to support the findings of this study are demonstrated in the manuscript. Further information on request is acquirable from the corresponding author Guanyu Zhang.
